# Supplementary material for: Are carbon emissions trading and green financial instruments synergistic? -Comprehensive quantitative research based on content analysis
Source: PLoS One. 2024 Mar 7;19(3):e0298601. doi: 10.1371/journal.pone.0298601 (PMC10919601; doi:10.1371/journal.pone.0298601)
Supplement: S1 Data — (ZIP) [file pone.0298601.s001.zip › Data/地级市Z_F工作报告.zip!/╝¬┴╓/│ñ┤║╩╨/└┤╫╘╣1⁄2╓┌║┼í╛┬φ┐╦╩2╛▌═°í┐_2003.docx]

各位代表：

　　 现在，我代表市人民政府向大会作工作报告，请予审议；并请列席会议的同志提出意见。

　　 一、五年工作回顾和二○○二年的简要总结

　　 自1998年市十一届一次人民代表大会至今的五年来，我们认真贯彻党的十五大、十六大精神，在省委、省政府和市委的领导下，团结全市各族人民，抢抓机遇，开拓前进，圆满完成了本届政府各项任务，实现了跨越式发展的阶段性目标。

　　 ——国民经济持续快速增长，综合经济实力显著增强。国内生产总值等5项主要经济指标实现翻番。预计2002年，全市国内生产总值达到1150亿元，超本届政府计划150亿元，年均增长13%，占全国经济总量的比重由1997年的0.73%提高到1.14%。全口径财政收入107.5亿元，超计划22.5亿元，比1997年增长1.2倍；五年累计实现财政收入404.8亿元，年均增长16.6%。规模以上工业完成产值1120亿元，比1997年增长1.6倍，年均增长20.6%，在15个副省级城市中的位次由1997年的第13位上升到第7位。固定资产投资五年累计完成1176亿元，超计划176亿元，年均增长24.7%。外贸进出口总额完成25亿美元，比1997年增长1.5倍，年均增长19.7%。

　　 ——结构调整成果显著，经济整体素质全面提升。三次产业比重发生较大变化，一、二、三产业增加值占国内生产总值比重由1997年的21.1∶41.5∶37.4调整到12.8∶45.4∶41.8。效益型农业发展迅速，以大成、德大、皓月集团为代表的农业产业化龙头企业发展到120户，畜牧业占农业总产值比重达到51%。工业经济整体素质和市场竞争力明显增强，成为拉动经济增长的主要力量。规模以上工业企业发展到577户，五年累计完成新产品产值860.8亿元；一汽等30户重点企业实现产值占全市比重83.9%；汽车、食品、光电子信息、生物与医药四大主导行业占全市比重88.1%；高新技术产业产值达到400亿元，比1997年增长4.7倍。五年累计企业技术改造投资217.7亿元，比前五年增长1.1倍。第三产业结构进一步优化，金融保险、旅游等行业蓬勃发展。所有制结构调整成效突出，混合型经济发展迅速，非国有经济占国内生产总值比重达到60%，比1997年提高19个百分点。

　　 ——各项改革不断深化，社会主义市场经济体制初步建立。深入开展国企改革攻坚战，较好完成了国有及国有控股大中型企业三年脱困阶段性目标。全市121户中型以上国有企业80.2%改为公司制，1057户中小企业73%完成改制。资本运作成效突出，在深、沪两地及香港上市公司21家，总股本78.4亿股，累计募集资金130.2亿元。以养老、失业、基本医疗保险为重点的社会保障体系初步建立，74万名职工参加失业保险，40万名职工参加基本医疗保险，90万名职工和离退休人员参加基本养老保险，基本养老金社会发放率97.6%。建立和完善了城市居民最低生活保障制度，五年累计投入保障资金14424万元，受保人数14.7万人。住房、人事、分配制度和投融资体制等项改革逐步深入，要素市场不断完善，中介服务机构迅速发展，市场在资源配置中的基础性作用日益增强。较好地完成了各级政府机构改革任务。

　　 ——开发区经济迅猛发展，对外开放取得新成就。以招商引资为重点，全面扩大对外开放，拓展了经济发展空间。2002年，实际利用外资6.2亿美元，比1997年增长2.6倍；五年累计引进外资20.7亿美元，引进内资420亿元。外商投资企业2600多家，其中大众、伊藤忠等28家世界500强企业落户我市。大力开拓国际市场，外贸进出口总额占国内生产总值比重达到17.9%。同我市有贸易往来的国家、地区，比1997年增加了80个；文化等其他领域的对外交流与合作日益扩大，外事、台务、侨务等项工作在对外开放中发挥了积极作用。作为对外开放“窗口”的开发区，保持了强劲发展势头，2002年，五个开发区实现国内生产总值362.7亿元，比1997年增长2.9倍，占全市国内生产总值比重达31.5%。

　　 ——城市建设步伐加快，环境质量明显提高。突出“经营城市”理念，坚持可持续发展战略，不断拓展城市发展思路和空间。五年累计投入资金300多亿元，实施了一大批城市基础设施工程。“引松入长”二期、石头口门水库增容等源水建设工程的建成使用，缓解了连年干旱造成的“水荒”压力，为人民生活和经济发展提供了有效保障。绕城高速公路、轻铁一期工程及亚泰大街等60多条街路的建成使用，不仅有效地缓解了城市快速运转的“瓶颈”问题，而且使城市的骨架进一步完美、特色更加鲜明。伊通河风光带工程的实施，以及环城绿化带一期工程和牡丹园、长春公园等10个开放式公园的投入使用，进一步改善了城市环境质量，大大提升了城市的品位和层次。“一级规划、二级建设、三级管理”体制的实施，以及“畅通工程”、“取缔马路市场、禁止马车进城”、拆“三小”等多项综合整治活动的开展，使城市管理水平迈上了新台阶。现今，我市城市综合气化率94.5%，垃圾袋装化覆盖率82%，源水水质合格率100%，污水处理率66.8%，城市绿化覆盖率39%，空气质量基本达到国家二级标准，成为中国内陆生态环境质量最好的城市之一。

　　 ——关心群众切身利益，人民生活质量不断提高。2002年，城市居民人均可支配收入和农民人均纯收入6900元和3147元，分别比1997年增长46.7%和38%；城乡居民储蓄存款由386亿元上升到710亿元，年均增长13%，全市人均储蓄超过10000元。在收入增加的同时，人民群众的消费水平不断提高。全市社会消费品零售总额完成402亿元，比1997年增长86.3%，年均增长13.2%；城乡居民人均消费支出由3173元提高到5702元，年均增长12.4%；城市和农村居民恩格尔系数分别由1997年的50.4%、51.2%下降到39.7%和47%。城乡人均住房建筑面积分别为21.2平方米和18.5平方米。人口自然增长率控制在3.28‰以内。城乡居民生活总体达到小康水平。社区建设稳步推进，全市各类社区服务网点已达8000多个，社区卫生服务覆盖人口90%以上。大力实施“再就业”工程，五年累计实现再就业25万人（次），城镇登记失业率控制在3%以内。把解决群众的难点问题摆在政府工作突出地位，认真扎实为群众办实事。从薄弱校改造、农村改水改厕、“菜篮子”工程到大气环境治理、伊通河改造，五年（列入《政府工作报告》中）共完成实事工程60件、221子项，件件关联群众切身利益。

　　 ——科技教育与各项社会事业协调发展，精神文明建设全面推进。“长春·中国光谷”高标准起步，2002年，全市光电子信息产业完成产值90亿元，比1997年增长2倍，光电子信息企业已发展到220户。生物与医药行业迅速扩张，完成产值40亿元，比1997年增长近4倍，生物与中药现代化企业发展到89户。完成了长春信息港建设工程，政务信息化、社会公众信息化和企业信息化发展迅猛，信息化带动工业化的进程明显加快。把教育摆在优先发展的地位，全市五年教育经费投入74.9亿元，年均增长15.2%。强化素质教育，中小学办学体制和学校内部管理体制改革取得明显成效。突出农村教育和职业教育的战略地位，深化体制改革，强化结构调整，产生了积极的社会效果。长春职业技术学院成为全国职业教育品牌学校。市属医疗卫生体制改革取得实质性进展，食品卫生三年整顿任务基本完成。新闻、出版、文化、体育等各项社会事业全面发展，广电中心、国际会展中心、五环体育馆等一批文化体育基础设施建成投入使用。积极实施人才战略，市场配置人才机制基本形成，五年培训各类专业技术人才17.4万人（次），引进各类急需短缺人才1.1万人。多种形式的群众性文明创建活动蓬勃发展，社会文明程度不断提高，首批进入全国创建文明城市先进城市行列，四次荣获“双拥模范城”光荣称号。民族宗教工作取得新成绩。

　　 ——实行政务公开，坚持依法行政，民主法制和软环境建设取得新成绩。坚持不懈地开展软环境建设，结合长春实际，不断赋予软环境建设新的内涵，全力打造良好的长春“法制环境、服务环境、政策环境、社会环境”。“学习经济开发区海关活动”和建委等6部门试点工作取得良好成效；加快了软环境载体建设，启动基本建设等4个服务中心，成立长春政务中心，增强了政府工作的透明性。进一步改革审批制度，努力扩大创业自由度。报请市人大常委会批准废止的地方性法规5件，废止政府规章和规范性文件371件。审批、核准、备案事项由932项减少到465项，减幅为50.1%。廉政建设和反腐败斗争取得新成果。自1999年成立政府采购中心以来，组织公开招投标采购109次，节约财政资金9400万元。主动接受人大的法律监督、工作监督和政协的民主监督，认真办理人大议案、建议、意见和政协建议案、提案，努力扩大社会各界的知情权，提高政府民主决策、科学决策水平。高度重视社会稳定工作，认真处理人民群众来信来访，积极化解矛盾，努力解决人民群众的困难。“12345”市长公开电话自1999年开通以来，共受理群众来电374485件，办结354263件，办结率达94.6%，一大批群众关心的难点热点问题得到解决。深入开展“严打”整治和打击“法轮功”邪教组织斗争，强化社会治安综合治理，保持了政治稳定、社会安定。

　　 2002年是本届政府的最后一年。面对入世新形势和严峻挑战，全市上下团结一心，善为实干，坚持加快发展不动摇，推进改革开放和现代化建设再上一个新台阶。与上年相比，国内生产总值增长13.1%，全口径财政收入增长10.4%，城市居民人均可支配收入和农民人均纯收入分别增长8.8%和13%，为实现跨越式发展目标奠定了坚实基础。一是坚持奋发有为，集中全力扶强做大优势产业，努力赢得市场竞争主动权，开创了工作新局面。农业产业化骨干企业不断发展壮大，全市新建续建投资规模3000万元以上的农业产业化龙头项目22个，大成、德大集团均实现翻番目标。工业经济实现速度与效益同步增长，规模以上工业增加值增长17.7%，利润增长28.8%，工业效益综合指数达到170.2，位列15个副省级城市第一；特别是汽车工业再创新高，一汽集团汽车年产量突破50万辆；“解放”中重型卡车实现销售量20万辆，单品牌超过德国奔驰而跃居世界首位。二是坚持实事求是，创新工作方式方法，努力探索符合长春实际的经济工作新思路。在农业结构调整中，因地制宜开展了“两打”工程：一年打井20800多眼，超过建国50年来的总和；引导鼓励65万农民外出打工，比上年增长一倍多。有效地缓解了制约农业发展的水源不足及资金匮乏两项主要矛盾。在招商引资工作中，紧密结合长春的地缘、人文和气候等实际，巧打“三张牌”（大中华牌、欧美日韩牌、国内发达地区牌）、突出“三条线”（韩国、日本、台湾），有效地推动了工作开展，实际利用外资、引进内资分别比上年增长24%和14%。三是坚持迎难而上，集中主要精力解决主要矛盾，一些困扰多年的“老大难”问题实现了新突破。通过艰苦努力，恢复建设“半截子工程”25.4万平方米，确籍“无籍房”120万平方米，安置历史遗留的超期回迁居民531户；通过有效的市场运作，综合改造“长江路”，使之焕发生机，成为集旅游、观光、商贸于一体的商业街。四是坚持精品意识，高标准打造独具特色的城市品牌，着力提高城市的知名度和影响力。通过锲而不舍的努力，成功赢得2007年第六届亚冬会举办权，展示了城市魅力和实力，吸引了世界目光，凝聚了人心，架起了又一座长春通向世界的桥梁。同时荣获“国家园林城市”和“国家环保模范城市”称号，增加了城市无形资产的含金量，标志着城市生态环境质量和综合影响力显著提升。农博会、电影节、教育展再获圆满成功，成为全国具有重要影响的主题展会，提升了城市人气，创造了巨大商机。五是坚持以人为本，努力使人民生活向更全面更高水平的小康社会迈进，人民的物质和精神生活更加丰富。在初步实现小康的基础上，人们不再满足于基本生存条件，更加注重享受高科技和信息化带来的便捷和幸福。城市移动电话普及率每百人37.2部，每万人拥有私人轿车100辆、电脑1400台，日均“上网”32万人（次），年出境2万人（次），人均体育用地2.07平方米，超过国家规定标准1倍多。人们追求新时尚和新观念，精神生活愈加富足，市民娱乐、文化教育支出占消费性支出比重达到12.1%。与此同时，政府开始注重增加享受型设施投入，伴随着世纪广场、朝阳公园等一批公众活动场所的投入使用，以及市民健身中心和健身公园、社区健身路径的集中建设，使市民拥有了更多的公共娱乐休闲场所和设施。六是坚持用先进文化统领各项社会事业，努力实现“两个文明”相互促进、共同发展。自觉把发展社会事业作为建设先进文化的重要内容，不断加大基础设施投入。修复、重建了文庙、道台府、伪满皇宫等历史文物，挖掘和丰富了城市的历史文化内涵。多处老年活动中心和老年公寓的建成，以及市区主要街路盲人步道和无障碍通道的设置，弘扬了良好的社会道德风尚和时代精神；“代理妈妈”对4500名贫困儿童的认领，5000多名残疾人的就业安置，以及“慈善救助一日捐”1000多万元善款的筹集，都证明了长春“相互关爱、充满爱心”的良好社会氛围正在形成。

　　 各位代表，过去的五年是长春历史上发展最好的时期。社会主义市场经济体制实现了由局部改革到形成基本框架的整体性推进，城市基础设施建设实现了由严重制约到基本适应的本质性变化，经济发展实现了由速度不稳、效益不佳到持续高速增长、效益同步提高的实质性转变，人民生活实现了由温饱到小康的阶段性跨越。这些成绩的取得，是我们全面贯彻落实中央、省各项方针政策和重大部署的结果，是市委正确领导和市人大、政协有效监督的结果，是全市广大干部群众同心协力、顽强拼博的结果。五年来，海内外朋友特别是长春籍人士、港澳台同胞及外埠工商人士热诚关心、支持、帮助长春的建设和发展；中央、省驻长单位，中国人民解放军和武警驻长部队指战员，为长春两个文明建设做出了巨大贡献。在此，我代表市人民政府表示崇高的敬意和衷心的感谢！

　　 在肯定成绩的同时，也要清醒地看到，与国内先进城市相比，从市场竞争的压力和群众的期望上看，我们还有很大差距，发展中仍然存在许多困难和问题，主要是：市场经济体制尚不健全，要素市场不适应生产力发展需要；经济总量偏小，地方可用财力比重偏低；经济结构不尽合理，调整优化任务十分艰巨；城市化水平不高，县域经济发展任务繁重、财力不强；出口拉动力度不足，投资率偏低；就业再就业工作压力很大，城乡居民收入增长缓慢，部分群众生活困难；政府职能转变需要进一步加强，机关工作效率有待进一步提高；廉政建设、社会治安和安全生产等方面的问题也不可忽视，等等。对这些问题，我们要高度重视，采取有效措施，着力加以解决。

　　 二、今后五年的指导思想、奋斗目标和主要任务

　　 今后五年，是我们认真贯彻党的十六大战略部署、全面建设小康社会的重要时期。任务光荣而艰巨，形势复杂而多变。我们必须把跨越发展作为富民强市的第一要务，增强信心，奋发有为，抢抓机遇，超常运作，继续以高于全省、全国平均发展水平，高于大多数副省级城市的增长速度，加快长春发展进程。

　　 今后五年政府工作总的指导思想是：高举邓小平理论伟大旗帜，认真贯彻“三个代表”重要思想，全面落实党的十六大精神，按照省八次、市十次党代会的部署，以全面建设惠及长春700万人民的小康社会为目标，以加快发展为主题，以结构调整为主线，以改革开放和体制、机制、技术创新为动力，继续实施依法治市、科教兴市、开放带动、县域突破、可持续发展“五大战略”，加速建设现代制造业、区域性物流、科教文化“三大中心”，提高综合经济实力，促进物质、政治、精神“三大文明”协调发展，使长春早日进入全面、更高水平的小康社会。

　　 全市经济和社会发展的主要预期目标是：国内生产总值年均增长12%左右，2007年达到2000亿元，提前实现十六大提出的人均国内生产总值3000美元的奋斗目标；全口径财政收入超过185亿元，力争200亿元；全社会固定资产投资累计实现2000亿元。城市居民人均可支配收入超过9500元，力争10000元；农民人均纯收入超过4500元，力争5000元。城镇累计新增就业岗位35万个以上，城镇登记失业率控制在4%左右，农村外出务工经商人数占农村劳动力比重40%以上。完成上述目标任务，我们要努力做到“发展要有新思路，改革要有新突破，开放要有新局面，各项工作要有新举措”。

　　 ——以工业化思维谋划农业，做大做强工业，调整提升服务业，推进产业结构优化升级。全面建设小康社会关键在农村，要立足于农业增效、农民增收、财力增强，大力发展现代农业。以市场为导向，加快农业结构调整。重点发展种源农业、生态农业、创汇农业，连片建设800万亩专用原料基地、500万亩经济作物基地。充分依托粮食资源优势，集中发展优质肉牛等五大牧业体系，畜牧业产值占农业产值比重达到60%。推进农业标准化建设，农产品全部实现无害化、60%实现绿色化，率先建成全国农业标准化示范区。实施农村富余劳动力转移工程，形成以非农产业为主的就业结构，拓宽农民增收渠道。坚持用工业化思维谋划农业，新建扩建100项以上产业化大项目，基本形成布局合理、特色鲜明的县域工业群体。2007年，县（市）经济总量占全市比重稳定在30%，县（市）财力基本实现翻番。以改造传统产业和加快高新技术产业化为主线，狠抓汽车、食品、光电子信息、生物医药四大主导行业，建设大项目，培植大企业，走具有长春特色的新型工业化道路。举全市之力支持一汽实施“第三次创业”，培植大成、华禹光谷等一批起骨干支撑作用的大企业，建设100万辆汽车等一批具有突破性带动作用的重大项目。通过五年的努力，把长春建成真正意义上的国际汽车城、国内一流的绿色食品城、全国著名的光电子信息产业基地、区域性的生物医药和中药现代化产业基地。2007年，四大主导行业的销售收入突破2000亿元，销售收入超10亿元的企业达到50户，上市公司45户。围绕建设区域性物流中心，调整完善大商业、大市场、大流通格局，促进传统服务业向现代服务业转变。启动建设汽车贸易开发区等一批物流园区，积极培育和扶持以第三方物流为重点的现代物流企业，转型提升、整合重组现有物流资源，逐步构建功能完善、开放性的现代化物流服务体系。不断完善商贸、旅游、信息、会展四大服务功能，增强区域性中心城市辐射带动能力。适应居民消费结构变化，着力发展中介、咨询、教育培训、文化服务、体育健身等新兴服务业，培育新的经济增长点。到2007年，汽车贸易开发区要建成东北亚最大的汽车物流中心，全市各类物流园区销售收入达到400亿元，服务业增加值年均增长13%以上，服务业从业人员比重提高到50%。

　　 ——以体制创新为核心，全面推进各项改革，增强经济发展活力。毫不动摇地巩固和发展公有制经济，基本完成国有经济战略性调整和国有企业战略性改组，积极推行股份制，发展混合所有制经济。国有大中型企业要继续实行规范的公司制改造，完善法人治理结构，建立现代企业制度，绝大多数企业真正成为具有较强竞争力的市场主体。毫不动摇地鼓励、支持和引导非公有制经济加快发展，要把各种积极因素全部调动起来，使各种经济主体都活跃起来，放手让一切劳动、知识、技术、管理和资本的活力竞相迸发，让一切创造社会财富的源泉充分涌流，以造福于长春人民。鼓励一部分人通过诚实劳动、合法经营先富起来，一切合法的劳动收入和合法的非劳动收入都要得到保护，让广大群众放心大胆地创造财富。2007年，非公有制经济占全市经济总量的比重达65%以上。深化分配制度改革，扩大中等收入者比重，提高低收入者收入水平。深化人事制度、住房制度、投融资体制和医疗制度等各项改革，使改革向更深层次、更广领域全面推进。在更大程度上发挥市场机制在资源配置中的基础性作用，健全统一、开放、竞争、有序的现代市场体系。推进资本市场的改革开放，发展产权、土地、劳动力和技术等要素市场，创造各类市场主体平等使用生产要素的良好环境。

　　——以招商引资为重点，更加广泛地参与国际经济技术合作与竞争，全方位实现对外开放。强化开放意识，树立世界眼光。结构调整要顺应全球市场需求，新上重大项目要在国际产业分工体系中找准位置。充分利用国内国际两个市场，优化资源配置，拓展发展空间。外贸进出口总额达到55亿美元，外贸依存度每年提高1个百分点以上。有效利用国内国际两种资源，动员全市各个层面齐力抓招商引资，同时引进先进技术、智力和引管理方式。五年累计利用外资50亿美元、内资800亿元以上。鼓励和支持外资投资农业、制造业和高新技术产业，逐步推进服务领域开放。扩大科技、教育、文化等领域对外交往渠道，搭建开放发展的国际平台。实施“走出去”战略，积极到境外承包工程、办企业，开展劳务合作，带动产品、劳务和技术装备出口。密切关注、积极参与图们江地区开发。继续推进开发区“二次创业”，努力实现体制、科技、产业、环境“四大创新”，把开发区建设成为机制新、环境美、多功能、现代化的新城区，完成由奠基期到规模扩张阶段的实质性跨越。2007年，五个开发区实现国内生产总值1000亿元，占全市经济总量50%左右。

　　 ——推进科技进步与创新，加快信息化进程，为生产力发展提供不竭动力。把经济发展建立在科技进步的基础上，大力发展高新技术和先进适用技术，提高科学技术在经济增长中的贡献率。突出抓好开发区各类科技园区和“孵化器”建设，打造“生物与医药”、“光电子信息”等高新技术发展平台，引进一批项目，集聚一批企业，培植长春“创新经济”高地。高新技术产业产值达到1500亿元。正确处理技术创新和引进、消化、吸收的关系，加强四大主导行业等领域的关键技术开发，形成一批具有自主知识产权的核心技术。坚持科技体制创新，实行产学研一体化，促进科技教育与经济紧密结合，提高科技成果转化率。大力推进国民经济和社会信息化，建设“数字城市”。依托长春信息港构筑全市网络平台交互体系，建立完善工业信息等十大应用系统，满足政府、企业、社会信息化的需求。加强信息化基础设施建设，全市通信光缆总长度达到10000皮长公里，光纤主干网传输速率100G，城区宽带骨干网入口覆盖率80%，计算机普及率每百户38台，互联网接入率73%以上。以信息化带动工业化，使工业化在高起点上迅速发展。在企业新产品研发、生产、经营、管理和决策过程中广泛应用信息技术，应用计算机辅助设计技术的企业比例超过70%。大力发展软件开发和信息服务业，全市软件企业达到500家，信息产业增加值300亿元。

　　 ——以中心城市为依托，加快城市化步伐，促进城乡一体协调发展。坚持“规划先行”原则和“积极而为，适度超前”方针，加强城市基础设施和环境建设。实施铁北、八里堡旧城区改造，启动南部新城和富锋、兴隆分团，依托高新技术开发区建设城市副中心，超前规划以长春新机场为依托的东部新城，初步形成多中心、组团式的城市格局，使“舒朗、大气、通透、开放”的城市风格更加鲜明。实施引气入长、城市新水源、污水处理、热电厂扩建、县乡公路等十大工程，城市综合气化率达到98%以上，日供水能力130万吨，污水集中处理率75%以上，能源交通等各种基本生产要素供给满足城市发展需要。实施市容综合整治行动计划，全面启动“半截子工程”，拆除违法建筑。主要街路基本改造成为标准化街路，二环路以内主次干道实现美化亮化，中心城区市容市貌达到国内一流水平。实施“蓝天、碧水、绿地、安静”工程，高标准建设贯通城区的伊通河生态风光带，加宽环城绿化带，整治西部串湖，恢复波罗泡湿地，年均人工造林10000公顷。综合治理水体、空气和噪声污染，使长春的人居环境优美、生态环境良好、适宜市民生活，成为世人瞩目的中国内陆最具特色的生态园林城市。充分发挥中心城市的聚集、辐射和带动功能，积极发展中小城市，择优扶持一批重点建制镇，形成布局合理、特色突出、功能互补的城镇体系。消除城市化的体制障碍，取消就业歧视性政策，引导农村劳动力合理有序流动，推进城市化步伐。2007年，城市化水平达到50%以上。

　　 ——大力发展社会主义文化，建设社会主义精神文明和政治文明，推动社会全面进步。以“三个代表”重要思想为统领，大力发展社会主义先进文化，培育与社会主义初级阶段基本经济制度相适应的思想观念，营造有利于人才发展创业的良好机制，形成“人人想干事业、人人能干事业”的良好社会氛围。加强社会公德、职业道德和家庭美德教育，弘扬奋发向上、朝气蓬勃、团结友爱的城市精神，树立自强不息、诚实豁达的长春人形象。支持文化产业发展，整合文化资源，加强文化基础设施建设，发展群众文化。加快发展教育事业，造就一大批高素质劳动者、专门人才和拔尖创新人才。开展全民健身活动，提高市民健康水平。完善公共卫生医疗服务体系，加快实现“人人享有卫生保健”目标。发展新闻出版、广播电视等各项事业，满足人民群众多样化精神需要。随着经济发展，不断增加城乡居民收入，拓宽消费领域，优化消费结构，促进发展型和享受型消费大众化，使长春老百姓由单纯过日子变为享受新生活。健全和完善社会保障体系，积极发展社会福利、社会救助事业，帮扶困难群众，使他们的基本生活得到保障，并逐步过上小康生活，努力使长春的每个角落都富起来。进一步转变政府职能，提高行政效率，降低行政成本，建立“行为规范、运转协调、公正透明、廉洁高效”的新型行政管理体制，建设服务型政府。深入实施“依法治市”战略，加强社会主义民主法制建设，提高依法行政水平，推进政府决策科学化、民主化进程。

　　 举办亚冬会，是长春发展史上的盛事，也是推进城市现代化进程的难得机遇。充分挖掘亚冬会拉动经济增长和促进社会进步的潜力，以亚冬会助发展。要在国家和省的大力支持下，充分运用市场机制和国际化运作模式，高标准完成亚冬会比赛场馆的建设与改造，建设一批交通、通信、市政、生态大型项目和公共建筑，全面提升城市综合服务功能和整体形象。要全市动员、全民参与，凝聚人心，鼓舞士气。2007年，长春人将向亚洲证明：亚奥理事会做出的重大抉择是一次明智之举，长春人民奉献给亚洲的一定是历史上最成功、最具魅力的一届冬季体育盛会。

　　 三、二○○三年工作安排

　　 2003年是新一届政府的起始年，也是全面落实党的十六大精神的第一年，开好头、起好步十分重要。在新的一年里，要继续坚持加快发展不动摇，坚持干是硬道理，努力实现新一届政府各项工作的良好开局，为长春今后五年的发展赢得时间，争得主动。主要预期目标是：国内生产总值增长13%左右，全口径财政收入增长11.2%，城市居民人均可支配收入、农民人均纯收入分别比上年增长7.5%和8%；新开发就业岗位6.5万个，城镇登记失业率控制在3.5%左右，农村外出务工经商人数突破80万人次。重点要抓好以下八项工作。

　　 （一）加快结构调整和产业优化步伐，提高经济整体竞争力

　　 坚持在发展中调整结构，在结构调整中加快发展，保持经济快速增长的良好势头。大力发展市场农业，提高农村工业化水平，推进县域经济实现新突破。一产增加值实现158亿元，增长6%。加快农业结构调整步伐，提高农业综合效益。种植业要继续压缩普通玉米，集中发展劳动技术密集型园艺作物，做大做强优势产品，促进特色乡镇上档次、上水平。突出抓好饲料牧草，加快形成粮、经、饲三元种植结构。经济作物、蔬菜、饲料牧草分别发展到400万亩、200万亩和20万亩。畜牧业在扩张总量的基础上，突出抓住良种选用、饲料开发、防疫检疫、规模饲养和深加工等五大环节，解决质量和安全问题，提高国际市场竞争力。畜牧业增加值增长12%。加快农业标准化进程，建立完善农产品质量标准、质量监督检测、质量认证和安全执法等“四大体系”，开展农副产品上条码、上标识、上包装工作，打好“绿色食品牌”。启动建设16个无公害或绿色农产品示范园区，无公害和绿色食品面积新增45万亩。推进农业产业化经营，提高农民进入市场的组织化程度。广泛吸引社会资本兴办产业化龙头企业，鼓励大型流通机构、能人大户领办各种新型农村合作经济组织。进入产业化体系的农户比例达到45%以上。大力发展农村劳务经济。每个乡（镇）都建立一个外派劳务输出基地，培训农村富余劳动力1000人，为农民外出务工经商提供信息、培训和权益保障服务。按照依法、自愿、有偿的原则，进行农村土地使用权流转，发展规模经营。巩固农村税费改革成果，确保农民负担明显减轻、不反弹，确保乡镇机构和村级组织正常运转，确保义务教育经费正常需要。发挥市场机制作用，加快四县（市）所在地和五棵树等10个重点乡镇建设，完善功能，聚集人口，繁荣小城镇经济。本着“多予、少取、放活”的原则，加强对农业的保护和扶持。继续实施农村“打井”工程，每个具备条件的自然屯都要有一眼电机井。加强林业生态体系建设，退耕还林还草5000公顷，完成“三北”四期防护林4000公顷，规划启动城区西部、北部防风御沙林工程。

　　 工业是长春经济实现跨越式发展的重心和主要着力点。要围绕发展壮大四大主导行业，全面推进工业结构调整，提升工业经济整体素质和市场竞争力。工业增加值增长14%左右。滚动实施“重大项目”工程，启动建设一汽客车等一批投资超亿元的工业大项目，东高大豆深加工等14个项目年内投产。坚持用高新技术改造骨干企业，突出抓好中重型车改造等25项投资超亿元的重点技改项目，全年完成技改投资70亿元，增长15.7%。全力支持一汽换型改造与合资合作，整体带动工业经济加速增长。紧跟一汽发展规划，重组整合汽车零部件行业，向系统化、模块化配套供货方向发展，加快融入世界汽车产业分工体系。引导中小企业围绕国内外大企业、大项目，搞好协作配套，年内组建冲压、锻造、模具等5个加工中心。中小企业完成产值640亿元，增长16.4%。加快新产品开发步伐。奥迪A4轿车等30种重点新产品年内投放市场，实现产值332亿元。高起点规划建设各类民营工业园区，吸引企业合理集聚。依托双丰等重点乡镇建设10个县（区）工业园，围绕德大、皓月等龙头企业规划一批农副产品加工小区。引导企业利用信息化技术改进产品设计和生产工艺流程，抓好重点行业和重点企业的信息化改造。一汽轿车公司齿轮厂等近百户制造业信息化示范企业全面普及CAD、CAM（计算机辅助设计、计算机辅助制造）技术。

　　 大力发展服务业，是经济结构战略性调整的重要内容。要加快发展现代服务业，推动传统服务业提档升级。第三产业增加值增长13%。加快汽车贸易开发区、东站、铁北三大物流园区建设步伐，积极引进国际物流企业，扶持大众物流、欧亚集团等第三方物流企业或专业物流配送中心扩展业务，加快区域性物流中心建设进程。按照合理布局的原则，启动建设蔬菜中心批发市场等9个大型专业批发市场，抓好亚泰购物中心等14个万米以上大型商业项目，其中7个项目竣工投入使用。发展新兴业态和社区商业，新增各类连锁店100家。全社会消费品零售总额增长13.4%。抓好旅游资源的深度开发和综合利用，完成伪满皇宫周边环境治理及主要景区的主体建筑复原。以净月冰雪节等活动为载体，全力开拓旅游市场。旅游总收入增长30%。开发会展资源，挖掘办展潜力，全年举办全国性大型展会10个以上。办好汽博会、中国汽车产业奠基50周年纪念大会、农博会、国际雕塑大会等大型展会，提高档次，扩大影响。坚持依法治税，确保财政收入稳定增长。继续树立过紧日子思想，集中财力保稳定、保发展。推行国库集中收付和会计集中核算管理，扩大政府采购范围和规模，提高财政资金的使用效率。继续抓好市政、公用工程材料设备公开招标采购，从制度上防止“工程腐败”。积极防范和化解地方性金融风险。

　　 （二）坚定不移地推进改革，提高经济市场化程度

　　 调整国有经济的布局和结构，改革国有资产管理体制，是深化经济体制改革的重大任务。积极推进国有企业产权制度改革，竞争性行业中的国有独资企业要加快国有资本退出步伐。年内市属14户中型以上国有独资工业企业、5户较大规模的国有独资商贸企业、50%的市以下小型国有独资企业，实现国有资本退出。加快市政公用事业单位改革进程，公交集团、自来水公司、煤气公司分别减亏1000万元以上，市政、环卫、园林绿化三支队伍的“事转企”工作迈出实质性步伐。支持企业境内外上市和已上市企业的再融资，境内外新上市企业分别达到2户以上，股票市场融资规模超过20亿元，并培育一批上市预备企业。非上市公司要积极实施国有股减持。实行改制的大中型企业，要以“责权统一、运转协调、有效制衡”为目标，规范法人治理结构，年内60%的企业建立较为完善的现代企业制度。调整国有资本管理、运营和监管体系，改革国有资产考核办法，强化激励和约束机制，促进国有资产科学运营。通过变现国有资产、盘活土地资源等办法，多渠道筹集资金，弥补改革成本缺口，妥善安置职工。支持中、省直国有企业加快改革步伐，加强对集体企业改革工作的指导。

　　 发展民营经济对于充分挖掘蕴藏在群众中的巨大潜力，加快生产力发展具有重要意义。全力支持和促进民营经济实现突破性发展，凡国家法律法规未予限制的领域都要向民营经济放开，凡符合国家产业政策的都要放手让民营企业发展，切实为民营经济创造公平竞争的良好环境。在产权明晰的前提下，支持民营企业以多种形式参与国有、集体企业的改制、改组和改造，推动多种所有制经济在机制上对接，经营方式上取长补短。营造崇尚“建设者有功、纳税人光荣”的社会氛围，激发群众自主创业热情。民营企业和个体工商户新增2000户和10000户，销售收入10亿元和亿元以上的民营企业分别发展到8户和80户，民营经济增加值和税收分别增长26%和22%。支持乡镇企业技术改造、结构调整和体制创新，增强发展活力。

　　 （三）坚持“引进来”和“走出去”相结合，努力开创对外开放新局面

　　 实施市场多元化战略，在稳定日本、韩国等周边传统市场的同时，深度开发俄罗斯、越南等新兴市场，并逐步向独联体和东盟其它国家延伸。实现外贸出口总额11.5亿美元，增长9.5%。坚持以质取胜，优化出口结构，巩固玉米出口，扩大蔬菜、肉类等农副产品及精加工产品出口规模，努力把机电、高新技术产品培植成出口拳头产品。深化外经贸体制改革，推进外贸主体多元化。支持“三资”企业、自营出口企业组织出口，出口额各增长14%。按照国际市场需求组织生产，抓好采用国际标准和质量标准认证工作，年内60家企业通过相应的国际标准认证。积极地“走出去”，支持境外投资办厂，新办境外企业4户。扩大对外工程承包领域和规模，合同额增长10%。

　　 吸引外商直接投资，提高利用外资的质量和水平。组织各种灵活务实的招商活动，继续对日本、韩国、台湾地区开展团组招商；组织汽车零部件企业与大众、丰田配套体系内企业开展对接招商；以开发区各类园区为载体开展园区招商，配套引进资本、技术、管理、品牌和销售渠道。拓展引资视野，既要抓住上亿美元的大项目不放，形成引进一个项目、带来一批企业的集聚效应，也要引进一批有市场前景的中小企业。创新引资方式，既要吸引外商独资企业，也要鼓励跨国公司实施并购重组和支持外资企业增资扩股。实际利用外资7.5亿美元，增长21%。坚持内外并举，以发达地区的民营资本和国内著名厂商为重点，吸引外埠资本来长春投资兴业。实际利用内资140亿元，增长16.7%。

　　 开发区是拉动我市经济增长最活跃的力量。要全力支持开发区“二次创业”。按照“内部管理企业化、外部管理市场化”的思路，实施开发区体制和机制创新，加快建立“小机构、大社会”的管理体制和协调、高效的运行机制。按照“把管理寓于服务之中，把支持变成自身责任”的要求，全市各部门、窗口单位都要优化服务环境，为开发区快速发展做贡献、尽义务。各开发区要自我加压，鼓足干劲，为“二次创业”做出无私奉献。五个开发区年内引进超千万美元的大项目32个，实际利用内外资均增长20%；国内生产总值增长30%以上。

　　 （四）实施科教兴市战略，积极开发人才资源

　　 坚持引进与自主创新相结合，构筑科技创新体系，实现技术进步和产业升级的新突破。高新技术产业产值增长30%。集中力量和资源，着力抓好重大项目的开发、转化和产业化进程。生物医药行业要突出抓好水痘疫苗等10个重点产业化项目，加快天药科技等8个国家级新药研发中心建设步伐，产值比上年增长30%。光电子信息行业要集中扶持一批大企业大集团，白光二极管等7个产业化项目开工建设，镀膜材料等11个重点项目实现批量生产，产值增长35%。实施“特快列车”等七项政府引导计划，整合现有科技资源，大力推进官产学研结合。开展“市院合作工程”，实施可溶性导电聚苯胺等10个重大科研项目，激光彩色视频显示技术等5个项目进入中试阶段。鼓励企业与高校、科研院所共建博士后流动站、实验室、研发中心等各类科研机构，新建企业技术中心20个。推进经济和社会信息化进程。抓好宽带接入网建设，大中型企业、城区中小学校宽带接入率达到95%和90%，富豪花园等一批居民小区实现千兆到小区。全市政务信息网络系统投入运行，部分政务实现网上办理。

　　 教育是发展科学技术和培养人才的基础，必须摆在优先发展的战略位置。深化教育改革，促进基础教育、职业教育、成人教育和高等教育协调发展。加大农村基础教育投入，深化农村办学模式改革，控制农村中小学生辍学。加快高中教育优质化进程，新建两所寄宿制高中，15所示范性高中争取通过国家验收。整合职业教育资源，在四县（市）和双阳区各建一所职业教育中心。办好长春职业技术学院，组建职业教育集团。深化中小学内部管理体制改革，加强校长和教师队伍建设。支持在长高校扩大办学规模，探索在校特困大学生勤工俭学的扶持办法。继续推进“市民外语”工程，加强窗口行业从业人员外语培训。

　　 人才是第一资源。要加快实施人才战略，创造各类优秀人才脱颖而出的良好环境。支持知识、技术、经营管理等生产要素按贡献参与分配，出售专利所得、技术股权分红和经营管理股份分红等各种非劳动收入都要得到合法保护。认真兑现人才应享受的各项优惠政策和相关待遇，最广泛最充分地调动科研人员、企业家等各类人才创造财富的积极性。坚持以事业需要选人用人，实现人才使用“柔性化”。降低人才引进门槛，放活人才流动形式，年内引进短缺和急需人才3500人以上。

　　 （五）千方百计扩大就业，努力改善困难群众的基本生活

　　 就业是民生之本。扩大就业是当前和今后长时期重大而艰巨的任务。各级政府必须把改善创业环境和增加就业岗位作为重要职责，列入领导干部岗位目标责任制。全方位开发就业岗位，突出发展社区服务业等劳动密集型产业，全年开发社区就业岗位1.8万个；支持下岗失业人员以非全日制、临时性工作等方式实现多形式就业；积极开拓境内外劳务市场，国际劳务外派人员增长10%。落实促进再就业的各项政策，为下岗失业人员自谋职业和自主创业提供小额贷款；新吸纳一定比例下岗失业人员、并与之签订3年以上劳动合同的服务型企业，按比例减免相关税费。完善就业服务体系，建立市、县（市）区、街道、社区四级就业服务网络，形成齐抓共管的工作机制；加强职业技能培训，全年培训下岗失业人员1.5万人、新生劳动力1.6万人，企业在职职工培训率40%以上；搞好劳动力市场和职业介绍网络建设，为下岗失业人员谋职提供便利服务。引导下岗失业人员转变就业观念，鼓励自谋职业和自主创业。加强劳动用工管理，保障劳动者合法权益。

　　 高度重视和关心困难群众的基本生活。发展老龄事业，提高老年人福利服务质量。市老干部活动中心年内竣工并交付使用。依法安排残疾人就业，全年安置1920人。在大马路等五条街路修建盲人步道和无障碍通道，不断改善残疾人的生活质量。强化政府救济职能，发展社会福利事业，开展“经常性社会捐助”、“扶贫助困”活动，努力帮助困难群众解决重病就医和因贫失学等问题。落实居民最低生活保障制度，把符合条件的居民全部纳入最低生活保障范围，努力做到不漏保、不错保。确保国有企业下岗职工基本生活费、离退休人员基本养老金、失业人员失业保险金按时足额发放。继续扩大养老、失业保险覆盖面，做好非公有制企业职工养老保险和事业单位职工失业保险工作。养老、失业保险各新增参保人数5万人。稳步推进城镇职工医疗保险制度改革。

　　 （六）发展社会主义先进文化，建设社会主义精神文明，全力维护社会稳定

　　 牢牢把握先进文化的前进方向，推动社会主义文化的发展和繁荣。把弘扬和培育民族精神、城市精神作为先进文化建设重要任务，纳入公民教育和精神文明建设全过程，深入进行十六大精神和“三个代表”重要思想教育，贯彻《公民道德建设实施纲要》，引导市民树立正确的世界观、人生观、价值观和共同理想。积极开展群众性精神文明创建活动，推动文明城市建设深入发展。充分利用群众喜闻乐见的活动载体，发展特色群众文化。全年组织大型广场文化活动200场。繁荣文艺创作，多出文艺精品。加快文化基础设施建设，筹建长春艺术大厦，长春图书馆完成数字化改造，新闻出版大厦交付使用。推行全民健身计划，上半年全民健身中心向市民开放。承办好2003年亚洲汽车拉力赛等大型体育赛事。抓好疾病预防控制，传染病发病率控制在1.7‰以内。加强医疗垃圾无害化处理。启动建设北方肝胆医院。推进农村卫生基础设施建设，因地制宜发展农村合作医疗，改善农村医疗卫生状况。深化计划生育综合改革，全面实施妇女儿童发展规划，提高人口素质。人口自然增长率控制在5.2‰以下。启动新一轮地方志修志工作。深入开展军警民共建活动，巩固“双拥模范城”成果。认真做好民族、宗教和民兵、预备役工作。

　　 完成改革和发展的繁重任务，必须保持和谐稳定的社会环境。高度重视群众来信来访和人民调解工作，认真解决人民群众的实际困难，及时化解影响社会稳定的因素。加强社会治安综合治理，严厉打击各种刑事和经济犯罪，扫除“黄赌毒”等社会丑恶现象。切实加强生产、交通和消防安全管理，全面落实安全生产责任制，防止重大事故发生。继续深入开展同“法轮功”等邪教组织的斗争。高度重视民族团结，做好对西藏定结县的援助工作。

　　 （七）加快重点项目建设，增强经济发展后劲和城市承载能力

　　 重点项目是保持经济社会持续快速健康发展的支撑和依托，必须摆上重要位置，认真谋划和实施。深入开展“项目建设年”活动，继续紧跟国家积极的财政政策导向，启动一批国债项目，最大限度争取国债资金和贷款贴息支持。完成全社会固定资产投资365亿元，增长14%。挖掘储备项目，定期补充调整，建成动态、开放的项目库。推进投融资体制改革，开放城市基础设施、公用设施及科教文卫等投资领域。推行社会投资项目审批备案制，启动民间投资。完善土地收购储备制度，经营性国有土地使用权实行招标拍卖和挂牌出让，土地出让收益完成8亿元以上。

　　 继续加快城市基础设施建设。完善城市综合交通体系，方便市民出行。延伸景阳大街，打通基隆路，整修市区20余条主次干道，加快长吉南线、九德线等公路建设；启动轻铁净月线，实施54线有轨电车向铁北延伸工程；延长公交线路15条，更新大公共汽车245辆；长春新机场飞行区工程竣工，候机楼、航管楼实现主体封闭。推进住宅产业化，改善居民居住条件。年内房地产开发500万平方米，竣工260万平方米；放开和培育二手房市场，规范住房租赁市场；继续扩大住房公积金缴存覆盖面，提高使用率。搞好城市绿化及园林建设，提高市区绿化覆盖率。启动雕塑公园二期工程；伊通河城区中段综合整治有实质性进展，沿岸部分区域完成绿化美化；发挥市、区两级积极性，搞好区属公园综合整治，抓好养护管理，提高园林水平。城区新增绿地100公顷。继续抓好生态环境建设，巩固“国家环保模范城市”创建成果。加强空气和水污染治理，控制城区噪声污染；发展燃气用户2万户，新增集中供热面积200万平方米；热电二厂二期扩建工程完成厂房和配套设施建设。抓紧启动南部污水处理、雁鸣湖区域污水处理、北郊污水处理厂“中水回用”等项目。加强城市综合管理，提升城市形象。完成解放大路等五条标准街路和五个市容达标区建设；拆除违法建筑40万平方米；强化广场管理，提高市民休闲场所的档次和水平；搞好市容环境卫生，扩大垃圾袋装化覆盖面积，新建30座水洗公厕；继续抓好“畅通工程”建设，优化交通环境。依法调整和完善城市总体规划，编制完成城市近期建设规划、南部新城规划以及水系、交通等分团规划和专项规划；加强规划控制管理，推进规划管理法制化。改革城管监察体制，完善综合执法体系。

　　 抓紧亚冬会场馆、交通、通信、文化设施的规划设计，以及建设资金的筹措，集全市之资源拉开“办好亚冬会、建设新长春”的序幕。

　　 （八）建设“爱民、廉洁、务实、高效”政府，努力提高行政效能

　　 新一届政府的良好形象和行政效能，是完成各项任务的重要保证。按照 “爱民、廉洁、务实、高效”的要求，全面加强政府自身建设。

　　 进一步转变观念。坚持与时俱进，按照建设“学习型”政府的要求，大兴学习之风，勤于钻研，努力解放思想，转变观念，把思想认识从不合时宜的观念、习惯和体制的束缚中解放出来，把创新作为工作的常态，不断探索发展的新思路。政府全体公务人员都要振奋精神，鼓足干劲，以高昂的斗志锐意改革，以饱满的热情致力创新，以求实的精神促进发展。

　　进一步转变职能。按照建立“服务型”政府的要求，自觉把政府不该管的事务交给市场和社会，把直接从事的活动缩到最合理的范围，努力营造良好的经济发展软环境。继续推进审批制度改革，清理削减审批事项，规范审批行为。深入推行政务公开，增强行政透明度。严格依法行政，规范行政行为，对损害经济发展软环境的行政行为从严整处。

　　 进一步转变作风。认真践行“三个代表”重要思想，深入实际，体察民情，关心群众疾苦，努力为民解忧，始终保持与人民群众的血肉联系。继续办好市长公开电话，畅通政府与群众的联系渠道。力戒形式主义，老实做事，踏实干活，全身心地投入工作。各级领导要亲历亲为，靠前指挥，敢于碰硬，勇于解决难题。强化效率意识，加快工作节奏，推动政府工作全面提速。加强党风廉政建设，勤政为民，清廉为政。

　　 政府及其组成部门将一如既往地自觉接受市人大的法律监督和工作监督，自觉接受市政协的民主监督，自觉接受人民群众监督和舆论监督。

　　 根据群众的建议，2003年努力办好12件实事。

　　 1、实施再就业工程。对“4050”困难就业群体实施就业援助，年内9000人实现再就业。

　　 2、解决困难群众就医难问题。特困户、残疾人到市属医院（不含私营）就医，免收挂号费，门诊常规检查费优惠20%，住院床位费减半。

　　 3、建设第二回族公墓，设立回族殡葬服务站，完善设施。

　　 4、实施乡村公路建设工程，年内修建乡村公路400公里以上。

　　 5、开展“城乡手拉手、扶贫助学行动”，扶助农村薄弱学校建设，救助贫困学生完成学业；建设20个农村教育实践培训基地。

　　 6、实施街路综合改造和亮化工程。对影响市民出行的30条街路进行综合改造，配套搞好软硬覆盖；对二环路以内60条街道实施亮化。

　　 7、改造长吉高速公路和102国道（长平段）两个城市出入口，搞好绿化、亮化、美化。

　　 8、实施城区“二次供水”设施改造工程，年内综合整治“二次供水”设施305处，改造供水管线20公里。

　　 9、实施“三北”（北站房、北广场、北人民大街）续建工程，年内完成长春铁路客运北站房、北站前广场及地下停车场、南北广场连接地道建设，力争打通北人民大街。

　　 10、启动南湖公园三年改造工程，年内完成水体改造，实施部分绿化美化项目。

　　 11、改造二道区八里堡、宽城区铁北部分棚户区（面积30万平方米），改善市民居住条件。为城市低保家庭的住房特困户实物分配廉租房500 套。

　　 12、完善“菜篮子”产品质量卫生安全检测设施，逐步实行农产品市场准入制度，加强食品安全卫生质量控制。新增无公害蔬菜4000公顷。

　　 各位代表，让我们在党的十六大精神指引下，在省委、省政府和市委的领导下，团结一致，奋发拼搏，与时俱进，开拓进取，为建设一个繁荣富强、文明开放的新长春，为全市人民早日过上全面、更高水平的小康生活而努力奋斗。
